# Predictors of time to recovery from uncomplicated severe acute malnutrition among 6–59 months children treated in out patient treatment in health posts of Nagele Arsi district: a retrospective cohort study

**DOI:** 10.1186/s12887-022-03767-4

**Published:** 2022-12-13

**Authors:** Ashenafi Tsegaye, Bikila Lencha, Kebede Kumsa

**Affiliations:** 1 Basaku Health Center, Nagelle Arsi District, Oromia Ethiopia; 2 Department of Public Health, Madda Walabu University, Shashemene, Oromia Ethiopia

**Keywords:** Time to recovery, Outpatient therapeutics Program, 6–59 months, Nagele Arsi district

## Abstract

**Background:**

Access to outpatient therapeutic feeding programs (OTP) for all children who have uncomplicated severe acute malnutrition (SAM) remains a global public health priority. Identifying predictors that determine time-to-recovery from severe acute malnutrition optimize therapeutic success. However, reliable evidence on the determinants of time to recovery at health posts was not available in Nagele Arsi district of South Ethiopia.

**Objective:**

This study was aimed to identify determinants of time-to-recovery from uncomplicated SAM among children aged (6–59) months treated at an OTP in health posts of Nagele Arsi district, Southern Ethiopia.

**Methods:**

Institutional based retrospective cohort study was conducted among 357 children treated in Negele Arsi district from July1, 2018 to June 30, 2020. The children were selected using simple random sampling from 20 health posts. SAM treatment outcomes were compared against international SPHERE standards. The average time-to-recovery was estimated using Kaplan-Meier survival curve and the independent predictors of time to recovery were determined using multivariable Cox-proportional hazard model. The strength of the association was done using adjusted hazard ratio (AHR) with 95% confidence intervals. Statistical significance was declared at *p* value < 0.05. The results were presented by text, tables and figures.

**Result:**

A total of 284 (79.6%) children recovered during follow up. The mean weight gain for recovered children was 4.7 + 2.4 g/kg/day. The median time-to-recovery was 44 days 95% CI (42.7–45.3). Children who received Amoxicillin, AHR =2.574, 95% CI (1.879–3.525); de-wormed, AHR = 1.519, 95% CI (1.137–2.031); received Vitamin A, AHR = 2.518, 95% CI, (1.921–3.301) and new admissions, AHR = 1.823, 95%CI, (1.224–2.715) were more likely to recover. However, those who admitted with non-edema, AHR = 0.256, 95% CI, (0.189–0.346); had cough at admission, AHR = 0.513, 95 CI, (0.366–0.719) and had diarrhea at admission AHR = 0.5, 95% CI, 0.5 (0.350–0.712) were less likely to recover.

**Conclusion and recommendation:**

The recovery rate was within the acceptable ranges of International Sphere Standards. Those children who had cough and diarrhea should be given due attention from health extension workers and program planners. Appropriate provision of routine medication and timely intervention of co-morbidity are needed to increase chance of early recovery.

**Supplementary Information:**

The online version contains supplementary material available at 10.1186/s12887-022-03767-4.

## Introduction

Globally, approximately 49 million children under five were wasted and nearly 17 million were severely wasted in 2019 [1]. According to United Nations Children Fund (UNICEF), World Health Organization and World Bank group joint malnutrition estimate in 2018, about 14 million wasted children’s lived in Africa and four million were specifically found in East Africa [2].

Malnutrition contributes for 20% of pediatric hospital admissions and associated with more than half of all child deaths in Ethiopia through increasing the risk factors of other child illnesses [3, 4]. In Ethiopia, the prevalence of wasting decreased from 12% in 2005 to 7% in 2019 in the past 15 years [5] and severe wasting is estimated to be 3% [1].

Uncomplicated SAM is among many forms of malnutrition, specifically under-nutrition, which is defined as extremely low weight for height, and/or by the presence of nutritional edema. It is predominantly measured by one or more of the following criteria: weight-for-height (WFH) less than − 3 Z-scores; mid-upper arm circumference (MUAC) less than 115 mm for children (6–59) months and presence of bilateral pitting edema [6].

Outpatient therapeutic feeding program (OTP) is part of community-based management of acute malnutrition (CMAM) for children with uncomplicated severe acute malnutrition (SAM). The program services include diagnosis and provision of ready-to use therapeutic foods (RUTF) which contain abundant Iron, folic acid and vitamin A [7, 8].

First, children are identified by health extension workers using MUAC and edema criteria according to the national protocol [7]. An appetite test for children 6–59 months is performed every week and supplementation of medications like amoxicillin on admission of all SAM patients for 5 days, measles vaccine on the fourth week if the child has not yet received the measles vaccine for children 9–59 months and de-worming at 2nd week of treatment for children 24–59 months in OTP [7, 9, 10].

Studies conducted on OTP in different parts of Ethiopia revealed that the recovery rates of under five children with SAM ranged from 65 to 84% [11–15] and the median time to recovery ranged from 35 days to 61 days [11, 13, 14]. The determinants of time to recovery from SAM identified by previous studies were maternal illiteracy, delay in seeking care, supplementation of vitamin A, de-worming [11]; vaccination status; admission category [16]; routine medication [17]; presence of edema at admission [12]; type of SAM [18]; sharing of ready to use therapeutic food (RUTF), co-morbidity with malaria, anemia, diarrhea, cough and vomiting [19]; distance from health post [10]; and two and above years of age at admission [11, 20]. However, some of the findings were controversial and inconclusive. For instance, couple of studies from Ethiopia reported positive association between age and recovery from SAM [14, 17]. But, another study conducted in South Ethiopia reported negative association that those above 3 years were less likely to recover compared to their counterparts [13]. Further research with adequate sample size was mandatory to resolve the disagreement seen between studies.

Effectiveness of the treatment is evaluated by recovery rate, death rate, average length of stay, default and weight gain [21]. According to the SPHERE standard, acceptable level of mortality, recovery rate and default rate should be below 10%, above 75% and below 15%, respectively [21]. However, these parameters are still not achieved in many developing countries including Ethiopia [22]. Majority of the previous studies were conducted in hospitals and health centers. There is a dearth of literature at the health post level despite the fact that majority of the cases were treated there as an OTP. Therefore, our study was aimed to identify time to recovery and its determinants among 6–59 months children with uncomplicated severe acute malnutrition treated at an outpatient therapeutic program in Nagele Arsi Rural district, South Ethiopia.

## Methodology

### Study design, setting and participants

We conducted a retrospective cohort study in Nagele Arsi rural district located at 216 kms from Addis Ababa to South. Nagele Arsi rural district is one of the 13 districts of West Arsi Zone. It is bordered on the South by Shashemene Town, on the West by Shala district, on the North by Bulbula, on the North east by Heban Arsi district, and on the East by Kore district. The district has thirty-six kebeles and total population of 211,060, which are 34,679(16.43%) under five and 31,659(15%) are 6–59 months. The agricultural sector is very important for the livelihood of peoples in the district. Livestock and livestock products, crops produced in the district like barley, potatoes, maize, wheat and teff are commonly used for consumption and market purpose. The district is known by its local alcohol production which is called “Araqee” in local language. It is very labor intensive and used as the source of income for women in the district [Nagelle Arsi District Health Office, 2019/2020].

Nagelle Arsi was selected because of its high prevalence of severe acute malnutrition despite the fact that the district is one of the productive areas in West Arsi Zone.

The district has seven governmental health center and thirty-six health posts. Each health post has two health extension workers (HEWs) employed to provide a package of preventive and essential curative services including the management of uncomplicated SAM in children. HEWs identify SAM cases from their catchment area through multiple modalities including periodical growth monitoring and promotion, Routine health extension program (HEP), Community Based Nutrition (CBN) and Integrated Community Case Management (ICCM). According to Nagele Arsi district annual report (2019/2020), 901 children are admitted with uncomplicated SAM [Nagelle Arsi District Health Office, 2019/2020].

Health post-based retrospective cohort study was conducted using records of children with uncomplicated SAM who were treated on the OTP at health posts of Nagele Arsi district from July 1, 2018 to June 30, 2020.

### Inclusion and exclusion criteria

Our inclusion criteria were having middle upper arm circumference (MUAC) < 115 mm and presence of bilateral pitting edema (grade +, ++). We excluded uncomplicated SAM cases that had incomplete data on the charts.

### Sample size determination and sampling procedure

The sample size was determined for both objectives using single population proportion formula and double population proportion separately. The assumptions made for the first objective were proportion of recovery 65.3% from the study conducted in North Gondar Zone, North West Ethiopia [19]; the standard normal coefficient of 1.96 for 95% Confidence interval, desired degree of precision 5%. The estimated sample size was 348 which is higher than the sample size calculated for the second objective. In order to compensate for the lost and incomplete cards, the sample size was increased to 383.

**Fig. 1:**
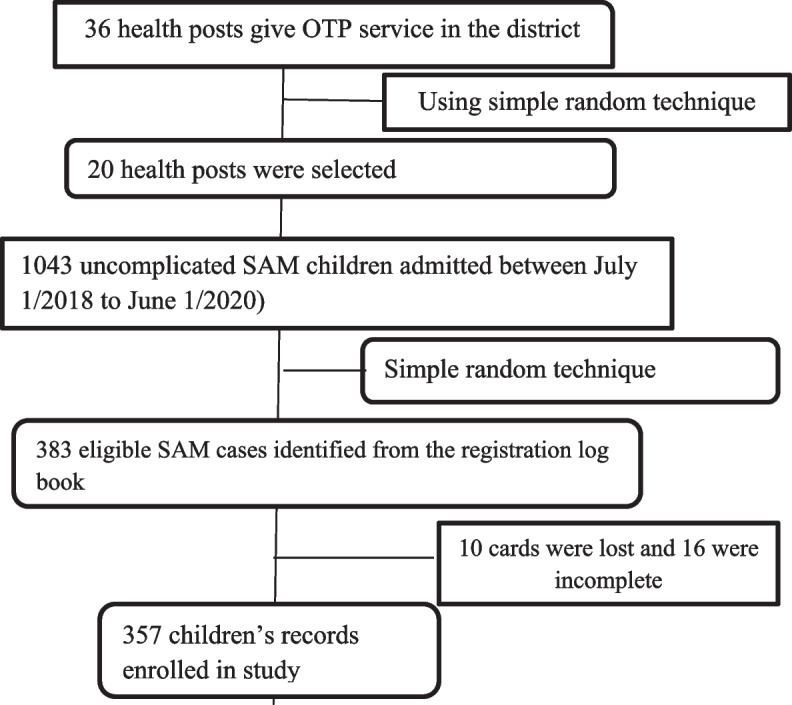
Sampling flow chart among children with uncomplicated SAM

In the study district, there are 36 health posts. Populations living around these health posts are assumed to be homogenous. As the result, 20 health posts were selected at random using lottery method (more than 50% of the total health posts). The sampling frame was prepared for each selected health post using eligible children with uncomplicated Severe Acute Malnutrition from the registration logbook. Then the total sample size was allocated to the selected health posts using probability proportional to size (PPS) of children with SAM. Finally, a simple random sampling was employed to select the participants of the study from each health post from the list of children medical registry number available at the respective health posts (Fig. 1).

### Data collection tool and procedure

The data extraction tool was adapted from Ethiopian protocol for the management of SAM and the sphere standard for the management severe acute malnutrition [7, 21]. The tool includes patient characteristics, anthropometric measurement, routine medication, follow up of consecutive weeks and outcome of the treatment at end of 8 weeks. Data extraction was done by five experienced nurses on management of severe acute malnutrition and supervised by two public health officers after 2 days of intensive training.

### Measurement of variables

Outcome variable of the study is time to recovery defined as number of days it takes from admission until a child is recovered from SAM. Recovery is defined as children who had no edema for at least 2 weeks or MUAC is ≥12.5 cm and they have had no edema for at least 2 week [7, 8]. Defaulter is a patient that is absent for two consecutive weeks (14 days) and confirmed by a home visit [7]. Death refers to the patient death and has to be confirmed by a home visit [7]. Censor refers to defaulter from treatment, transfer out, those who died with indirect and direct causes and those cases with unknown outcome at the end of the study period.

### Explanatory variables

Age of child is categorized as (6–23 vs 24–59), sex (male vs female), distance from health post in minute (≤ 30 vs > 30), edema (present vs absent), type of admission (new vs readmission), presence of comorbidities like cough, diarrhea, vomiting, skin infection, anemia, malaria and use routine medications like Folic acid, Albendazole/Mebendazole, measles vaccine, Amoxacillin and Vitamin A were extracted using yes/no response.

### Data quality management

The checklist was tested using 20 records in one health post outside the selected ones and necessary corrections were made before actual data collection. Training was provided to data collectors and supervisors on the data collection process, contents of the checklist and overall methodology of the study. The data checked for completeness and consistency and feedback was provided on daily basis for corrections.

### Data processing and analysis

Data was checked, coded and entered into Epi Data version 3.1software and exported to SPSS version 25 for cleaning and analysis. The patient cohort characteristics were described in terms of mean (Standard deviation) and median for continuous variables and frequency distribution and percentage for categorical variables. The following formulas were used to calculate recovery rate, average length of stay and weight gain.

Recovery rate was calculated as

$$\frac{number\ of\ patients\ discharged\ for\ recovery\ast 100}{total\ number\ of\ exits}$$

Average length of stay was calculated as

$$\frac{\textrm{sum}\ \textrm{of}\ \textrm{length}\ \textrm{of}\ \textrm{stay}\ \textrm{in}\ \textrm{hospital}\ \textrm{in}\ \textrm{days}\ }{\textrm{Number}\ \textrm{of}\ 6-59\textrm{months}\ \textrm{children}\ \textrm{cured}}$$

Weight gain (g/kg/day) was calculated as

$$\frac{\textrm{discharge}\ \textrm{weight}\ \textrm{in}\ \textrm{gram}-\textrm{admission}\ \textrm{weight}\ \textrm{in}\ \textrm{gram}}{\textrm{admission}\ \textrm{weight}\ \textrm{kilogram}\ast \textrm{number}\ \textrm{of}\ \textrm{days}\ \textrm{between}\ \textrm{minimum}\ \textrm{weight}\ \textrm{and}\ \textrm{discharged}\ \textrm{weight}}$$

Actuarial Life Table analysis was used to estimate cumulative proportion of recovery among children with SAM at different time points. SPHERE internationals standard was used for the comparison of SAM treatment outcomes [21]. Kaplan Meier Survival Curve was used to estimate nutritional recovery in days after initiation of outpatient treatment and log rank test was used to compare recovery curves for different categories of determinants.

Bi-variable and multivariable Cox-proportional hazard models were used to identify determinants of time-to-recovery. Those variables that have a *p*-value less than 0.25 in the bi-variable analysis were entered to model. The proportional hazard assumption of the model was assessed graphically by log- minus-log survival curve against time for different variables. Multicollinearity between explanatory variables were checked using variance inflation factor (VIF > 10) and no variables with multicollinearity were found. Both crude and adjusted hazard ratio (AHR) with the respective 95% confidence intervals (CI) were reported and interpreted to show the degree of association between time-to-recovery and the explanatory variables.

### Ethical consideration

Ethical approval was obtained from the institutional review board (IRB) of Madda Walabu University with Ref no of MWU/970/13 E.C. Permission letter to conduct the study was obtained from Nagele Arsi district health office with Ref no. WEF/841/2013 E.C. Confidentiality of information was maintained through avoiding any personal identifiers such as a name of child on the questionnaires during data collection. All methods were carried out in accordance with the principles of declaration of Helsinki. Informed consent from the subjects and or legal guardians was not sought as the study was fully retrospective review of the chart. Madda Walabu University IRB waived the informant consent with the Ref No of MWU/970/13 E.C. Finally, recorded data was kept safely by locking it in the locker and key of the locked was accessed only by principal investigator.

## Results

### Socio demographic and admission characteristics

Out of the total sampled 383 uncomplicated SAM children enrolled in OTP during the period of July1, 2018- June 30, 2020, 357 were analysed. The sixteen cards were discarded due to the incompleteness and cards of ten children were not found. The mean age in months of the children was 17.94(±11.94) months with nearly two thirds (65%) being younger than 24 months. More than half (54.6%) of them were breast-feeding. More than three fourth, (77%) of the children enrolled into the study had MUAC less than 115 mm. Average weight (SD) of the cohort at baseline was 7.1 ± 2.2 kg (Table 1).

**Table 1:** Socio-demographic, Routine medication and supplementation, Nagele Arsi distrcict 2020/21 (*n* = 357)

Variables	Category	Frequency	Percentage
Sex of Child	Male	176	49.3
	Female	181	50.7
Distance from Health post	< 30 minute	128	35.9
	> 30 minutes	229	64.1
Breast feeding	Yes	195	54.6
	No	162	45.4
Criteria to identify malnutrition	MUAC < 115 mm	275	77
	Edematous (+, ++)	82	23
Age categorized	0-23 months	232	65
	24-59 months	125	35
Type of Admission	New admission	313	87.7
	Readmission	44	12.3
Weight	Mean (standard deviation)	7.1(±2.2) kg	

### Clinical profile of children, routine medication and treatment

One hundred seventy-three (48.5%) had at least one type of co-morbidity at admission. Cough, 82 (23%) and diarrhea, 68 (19%) were the most common co-morbidities followed by vomiting 53 (14.8%), skin infection 39 (10.9%), anemia 30 (8.4%) and malaria 8 (2.2%). Three hundred twenty (89.6%), 252 (72%), 136 (38.1%), 75(21%), and 76 (21.3%) received folic acid, Amoxacillin, Vitamin A, Dewormed and measles vaccine at enrolment respectively (Table 2).

**Table 2:** Medical co-morbidity, routine medication and treatment characteristics Nagele Arsi distrcict, Ethiopia 2020/21

Variables	Category	Frequency	Percentage
Cough	No	275	77.0
	Yes	82	23.0
Diarrhea	No	289	81.0
	Yes	68	19.0
Vomiting	No	304	85.2
	Yes	53	14.8
Skin Infection	No	318	89.1
	Yes	39	10.9
Anemia	No	327	91.6
	Yes	30	8.4
Malaria	No	349	97.8
	Yes	8	2.2
Folic acid	Yes	37	10.4
	No	320	89.6
Albendazole/ Mebendazole	Yes	75	21
	No	282	79
Measles vaccine	Yes	76	21.3
	No	281	78.7
Amoxicillin	Yes	257	72
	No	100	28
Vitamin A	Yes	136	38.1
	No	221	61.9

### Survival experience

The participants were followed for a total of 15,557 person days (42.6 person years) of observation. The overall incidence of recovery was 12.78 with 95% CI of (11.36–14.33) per 100 children per week observed. The probability of survival at 21st, 28th, 35th, 42nd,49th and 56th days were 88, 77, 62, 39, 23and 15%, respectively (Table 3).

**Table 3:** Proportion of recovery during follow up period in Nagele Arsi district, Ethiopia 2020/21

Interval Start Time	Number Entering Interval	Number of censored	Number Exposed to Risk	Number of recovered	Probability of recovery	Probability of not recovery	Cumulative probability NR
0	357	0	357.000	0	0.00	1.00	1.00
7	357	3	355.500	0	0.00	1.00	1.00
14	354	1	353.500	14	0.04	0.96	0.96
21	339	6	336.000	27	0.08	0.92	0.88
28	306	3	304.500	38	0.12	0.88	0.77
35	265	1	264.500	52	0.20	0.80	0.62
42	212	7	208.500	76	0.36	0.64	0.39
49	129	1	128.500	53	0.41	0.59	0.23
56	75	12	69.000	24	0.35	0.65	0.15
63	39	39	19.500	0	0.00	1.00	0.15

*NR* Not recovery

### Time to recovery of children with uncomplicated severe acute malnutrition

A total of 284 children recovered from SAM, making it a recovery rate of 79.6% and the remaining 20.4% were censored. Reasons for censoring were: 46 (12.9%) were non-respondent, 19 (5.3%) referred to next level to seek better service. The proportion of death, defaulter and unknown were 0.3, 0.8 and 1.1% respectively. The overall median time-to-recovery was 44 days, 95%CI (42.7–45.3). The mean weight gain was 4.7 + 2.4 g/kg/day. Mean catch-up growth was 5.05 g/kg/day for recovered children enrolled with MUAC< 115 mm and 3.6 g/kg/day for edematous children.

**Fig. 2:**
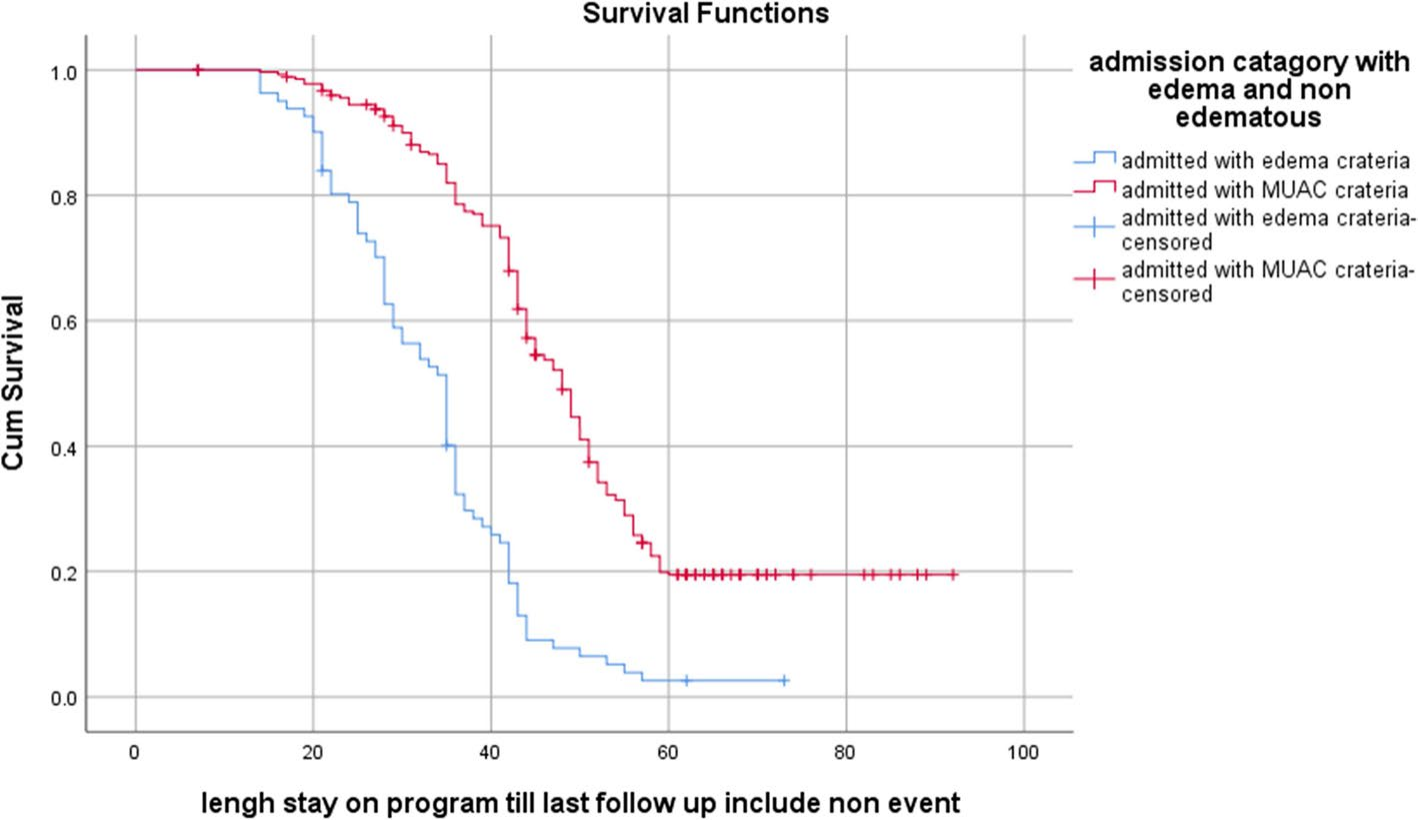
Kaplan Meier estimate of survival among children with uncomplicated SAM by admission criteria

**Fig. 3:**
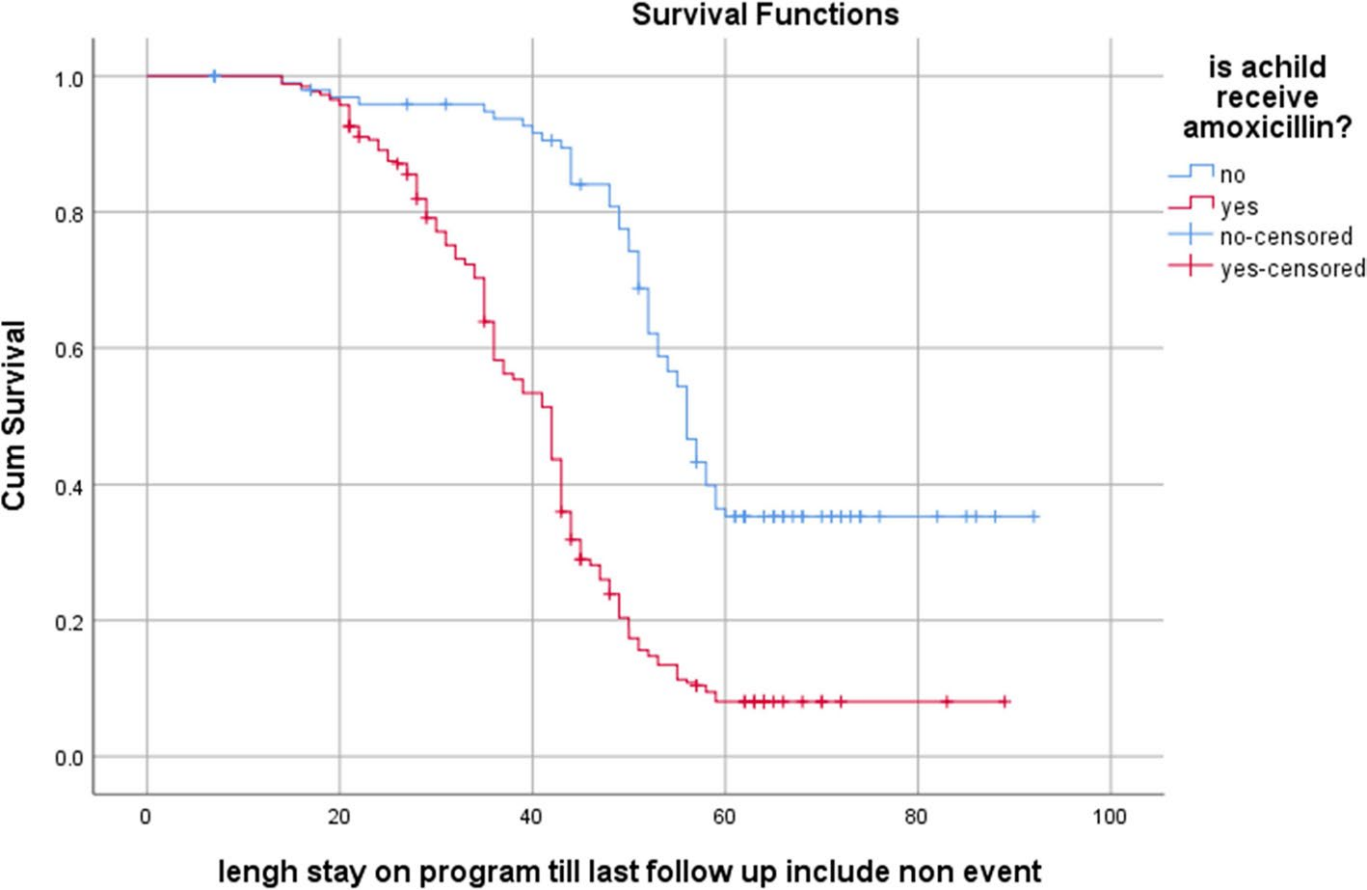
Kaplan Meier estimate of survival among children with uncomplicated SAM by Antibiotics administration

Kaplan Meier estimate of median recovery time showed that children who were diagnosed with non-edematous malnourished stayed longer before recovery than edematous malnourished counter parts (48 vs 35 days), chi-square = 89.3, *P*-Value < 0. 001 (Fig. 2). Similarly, children who did not receive Amoxicillin stay longer as compared with children received Amoxicillin with median recovery time of (56 vs 42 days) Chi-square 71.7, *P*-Value < 0.001 (Fig. 3).

### Predictors of time-to-recovery from severe acute malnutrition

Sex, age of the child at enrollment, time to travel from home to health post, type of admission, admission criteria, Co-morbidity with cough, vomiting, diarrhea, anemia and taking vitamin A, folic acid, de-worming and measles vaccine and Amoxicillin were candidate variables for multi-variable Cox regression model at *p*-value of < 0.25.

In the final multivariable Cox regression model, children who received Amoxicillin during admission were nearly 2.6 times (AHR =2.574, 95% CI (1.879–3.525) more likely to recover from uncomplicated SAM as compared with those who did not receive it. Children who received vitamin A were more than 2.5 times (AHR = 2.518, 95% CI, (1.921–3.301) more likely to recover from SAM as compared with those who did not receive it. Being dewormed had 1.5 times more likely to recover as compared to those who did not dewormed AHR = 1.519, 95% CI (1.137–2.031). Rate of time to recovery among children admitted for the first time nearly two times (AHR = 1.823, 95%CI, (1.224–2.715) as compared with readmission. However, children who were enrolled without Edema were 74.4% (AHR = 0.256, 95% CI, (0.189–0.346) less likely to recover from SAM as compared with edematous children. Children with diarrhea were identified 50% (AHR = 0.5, 95% CI, 0.5 (0.350–0.712) less likely to recover from severe acute malnutrition as compared with children without diarrhea. Similarly rate of time to recovery among those who had cough on admission delayed recovery time by 49.7% as compared to patient who had no cough AHR = 0.513, 95 CI, (0.366–0.719) (Table 4).

**Table 4:** Bivariate and multivariable Cox-regression analysis output in Nagele Arsi district, 2020/21

Variables	Categories	CHR (95%CI)	AHR (95%CI)	*P*- value
Age	0-23 months	0.797 (0.625–1.016)	0.909 (0.706–1.170)	0.457
	24-59 months	1		
Distance from health post	< 30 minutes	1.36 (1.07–1.73)	1.268 (0.991–1.621)	0.059
	> 30 minute	1		
Cough	No	1		< 0.001
	Yes	0.324 (0.237–0.442)	0.513 (0.366–0.719)**	
Folic acid	Yes	1.534 (1.055–2.229)	1.107 (0.735–1.668)	0.626
	No	1		
Diarrhea	No	1		< 0.001
	Yes	0.351 (0.252–0.492)	0.5 (0.350–0.712)**	
Anemia	No	1		0.161
	Yes	0.405 (0.254–0.647)	0.705 (0.433–1.149)	
Vomiting	No	1		0.053
	Yes	0.455 (0.324–0.64)	0.698 (0.485–1.004)	
Admission criteria	Edema Absent	0.295 (0.225–0.388)	0.256 (0.189–0.346)**	< 0.001
	Edema Present	1	1	
Vitamin A	Yes	3.272 (2.553–4.193)	2.518 (1.921–3.301)**	< 0.001
	No	1		
De wormed	Yes	1.668 (1.265–2.200)	1.519 (1.137–2.031)*	0.005
	No	1	1	
Amoxicillin	Yes	3.247 (2.420–4.356)	2.574 (1.879–3.525)**	< 0.001
	No	1	1	
Measles vaccine	Yes	1.187 (0.898–1.569)	1.101 (0.857–1.483)	0.527
	No	1		
Type of admission	New	1.412 (0.966–2.063)	1.823 (1.224–2.715)**	0.003
	Readmission	1		
Sex	Male	1.165 (0.923–1.470)	1.147 (0.899–1.464)	0.271
	Female	1		

1 = Referent category, * statistically associated *p* value < 0.05, ** statistically associated *p* value < 0.001

## Discussion

The study assessed time to recovery from uncomplicated SAM and its determinants among children aged 6–59 months treated at the health post of the Nagele Arsi district. The median time to recovery was 44 days 95% CI (42.7–45.3). It is in line with the study conducted in Shebedido district, Southern Ethiopia median time of recovery 42 days [13]. It is longer than the international standard (SPHERE) of 36 days set for the management of uncomplicated severe acute malnutrition; study conducted in North Gondar zone 38.5 days [19] and Shebedino Southern Ethiopia, 36 days [1]. The reason could be not following the management protocol for SAM; 39 (10.9%) children are allowed to stay on the program for more than 2 months. Additionally, edematous malnourished children accounted for less than one fourth (23% children’s) and there is also difference in magnitude of co-morbidity. On the contrary, it was shorter than the study conducted in Dire Dawa, Eastern Ethiopia shows 61 days [11] and study conducted in health posts of Arba Minch Zuria Woreda, Gamo zone 49 days [20]. A possible reason for this discrepancy could be related to the difference in magnitude of co-morbidity (lower prevalence of anemia and cough).

More than three fourth (79.6%) of the children were recovered in our study. This finding was similar with the study done in Shebideno district, 79.6% [1]; Dire dewa 79.8% [11]; Northern Ethiopia, 76.8% [23] and Southern Ethiopia, 78.7% [13]. The finding is in acceptable range of the sphere standard which states the recovery rate should be greater than 75% [21]. Our finding was higher when compared to study done in Kitui County Hospital, Kenya 73.3% [24]; Kamba district 67.7% [14]; Wolaita Zone, (68%) [12]. The observed discrepancies could be due to provision of relatively higher proportion Amoxicillin and vitamin A in our setting. But it was lower than study conducted in Bale, Oromia 84% [15] and 81.7% Gursum District of Ethiopia [18]. The observed discrepancies could be due to variation in timing and season in which the studies were conducted, availability and the accessibility of therapeutic foods and medications.

In our study, non-edematous children were less likely to recover from SAM as compared with edematous children. This finding is in agreement with other studies that were conducted in Shebadino district [1]. Similarly, a systematic review and meta-analysis done in Ethiopia found an association between no edema and recovery [25]. Rate of time to recovery among new admissions were two times more likely as compared with readmission. It is supported by study from North Wollo Zone which reported the likelihood of recovery was about 3.78 times higher among new admissions than those with re-admission [16].

Children provided with Amoxicillin at admission were more likely to recover compared with those not provided with Amoxicillin. This result was supported by studies from North Gondar zone, [19]; Afar Regional State [10]. A randomized double blind placebo controlled trial done among Malawian children reported similar findings [26]. Similarly, a systematic review and meta-analysis from SSA found an association between use of routine medication and rapid recovery [17]. This can be explained by the supportive effect of Amoxicillin in the treatment of infections and other complications associated with SAM [7].

Children who received vitamin A were more likely to recover from SAM as compared to their counterparts. This finding was in agreement with studies done in Amhara regional state [27]; Afar regional state [10]. Vitamin A is required for the integrity of epithelial cells in the body as well as in the maintenance of immune function. Therefore, vitamin A is essential to combat infections and the risk of illness and death from childhood infections.

Dewormed children were more likely to recover faster compared to those not dewormed. This finding is in line with studies from Dire dewa [11] and Jima Zone [22]. This could be due to high prevalence of intestinal parasite in severely malnourished children which results in reduced appetite and nausea in children who do not get de-worming.

In the present study children with diarrhea were less likely to recover compared to those without diarrhea. The finding is similar to studies from North Gonder Zone [19]; Arba Minch Zuria [20]; Sidama [1] and other study conducted in North wollo [16]. Diarrhea at admission is a negative predictor of time-to-recovery from SAM. The present study shows that diarrhea was found in one-fifth of children. Diarrhea is known to be more frequent in SAM cases due to the systematic immune-suppression effect and loss of the intestinal mucosal barrier due to malnutrition [28].

In the current study the rate of time to recovery among the children who had cough on admission delayed recovery time by 49.7% compared to the patient who had no cough. It is in line with a study from Gubalafto district, North Wollo Zone where recovery was 53% less likely among those with cough compared to their counterparts [16]. This might be due to the fact that SAM patient with co-morbidities requires a prolonged stay with an increased nutritional crisis and more nutrient requirement because of reduced appetite and nutrient absorption in comparison with their counterparts [29].

A study done in Amhara regional hospitals indicated that severely malnourished children having Anemia co-morbidity were 27% less likely to be cured as compared to those without anemia co-morbidity [27]. Anemia was a predominant factor that compromised recovery rate and increased mortality rate. There are also other studies that support the finding that being co morbid with anemia reduces the likelihood of recovery [4]. However, in this study Anemia co-morbidity is not a significant predictor of nutritional recovery time (*p* > 0.05). This may be due to practice in diagnostic capacity of anemia at health posts level by health extension workers.

The strength of this study was large sample size that enabled us to detect the difference with high precision and conducted at the lowest level (health post) of health system. More than 55% of the total health posts in the district were included.

However, the study has some limitations. Incomplete records were observed in some determinant variable and they were excluded. Potential bias due to excluded records and unknown status of defaulters were possible However, they were very small in number compared to recovered For diagnosis of severe acute malnutrition indices like weight for height/length at admission were not used. The diagnosis depends only on MUAC and edema criteria. Variables like maternal education status, paternal education status, family size, wealth index and food security were not studied. Other factors like dietary diversity score, perception of care givers on severity of SAM, RUTF sharing and selling behavior and perception on RUTF. Due to the observational design of the study, confounding from unmeasured variables cannot be entirely excluded. Thus, the findings of this study should be interpreted in consideration of this limitations.

## Conclusion and recommendations

The recovery rate was within the acceptable ranges of International Sphere Standards despite the higher median time to recovery among children. Type of admission, Admission criteria, deworming, antibiotics and Vitamin A supplementation and comorbidity were factors significantly associated with time to recovery. Special focus should be given for children admitted without edema and children admitted as readmission. A sound diagnosis and management of cases in OTP according national protocol needs special emphasis to supplementation of Albendazole/Mebendazole, vitamin A and provision of antibiotics for children. Early screening of co-morbidity like diarrhea, cough and anemia and timely intervention would increase the chance of recovery of children. Experiencing a lower rate of weight gain and a higher average length of stay among enrolled uncomplicated SAM patients were identified as major problems for the program effectiveness. However, as this was a retrospective study, the findings should be confirmed in another study carried out using prospective cohort study design.

## Supplementary Information

**Additional file 1.** English version of the checklist used to extract information.

## Data Availability

The datasets used and/or analysed during the current study are available from the corresponding author on reasonable request.

## Abbreviations

CBN: Community Based Nutrition
EDHS: Ethiopian Demographic Health Survey
ICCM: Integrated Community Case Management
MUAC: Mid Upper Arm Circumstance
OTP: Outpatient Therapeutics Program
PPS: Proportion To Population Size
RUTF: Ready To Use Therapeutics Food
SAM: Severe Acute Malnutrition
SPHERE: Social And Public Health Economics Research Group
UNICEF: United Nations Children Fund
WHO: World Health Organization

## References

[1] Teshome G, Bosha T, Gebremedhin S (2019). Time-to-recovery from severe acute malnutrition in children 6–59 months of age enrolled in the outpatient treatment program in Shebedino, Southern Ethiopia: a prospective cohort study. BMC Pediatr.

[2] UNICEF/WHO/World Bank Group Joint Child Malnutrition Estimates: Levels and trends in child malnutrition: key findings of the 2018 Edition. Geneva: World Health Organization; 2018. https://data.unicef.org/resources/jme. Accessed 5 Feb 2022.

[3] Fentaw R, Bogale A, Abebaw D (2013). Prevalence of child malnutrition in agro-pastoral households in Afar Regional State of Ethiopia. Nutr Res Pract.

[4] Wondim A, Tigabu B, Kelkay MM (2020). Time to recovery from severe acute malnutrition and its predictors among admitted children aged 6-59 months at the therapeutic feeding center of Pawi General Hospital, Northwest Ethiopia: a retrospective follow-up study. Int J Pediatr.

[5] EPHI I (2019). Ethiopia mini demographic and health survey 2019: key indicators.

[6] Guideline W (2013). Updates on the management of severe acute malnutrition in infants and children.

[7] health Fmo. In: Directorate MaCH, editor. Guidelines for the Management of Acute Malnutrition. Addis Ababa: Federal Ministry of Health Ethiopia (FMOH); 2016.

[8] ETHIOPIA GO: Guidelines for the Management of Acute Malnutrition NNP Implementation Guide 4. Addis Ababa: Federal Ministry of Health Ethiopia (FMOH); 2014.

[9] Bitew ZW, Alemu A, Worku T (2020). Treatment outcomes of severe acute malnutrition and predictors of recovery in under-five children treated within outpatient therapeutic programs in Ethiopia: a systematic review and meta-analysis. BMC Pediatr.

[10] Liben ML, Wuneh AG, Shamie R (2019). Factors associated with child survival in children admitted to outpatient therapeutic program at public health institutions in Afar Regional State, Ethiopia: a prospective cohort study. J Health Popul Nutr.

[11] Atnafe B, Roba KT, Dingeta T (2019). Time of recovery and associated factors of children with severe acute malnutrition treated at outpatient therapeutic feeding program in Dire Dawa, Eastern Ethiopia. PLoS One.

[12] Kabalo MY, Seifu CN (2017). Treatment outcomes of severe acute malnutrition in children treated within Outpatient Therapeutic Program (OTP) at Wolaita Zone, Southern Ethiopia: retrospective cross-sectional study. J Health Popul Nutr.

[13] Mengesha MM, Deyessa N, Tegegne BS, Dessie Y (2016). Treatment outcome and factors affecting time to recovery in children with severe acute malnutrition treated at outpatient therapeutic care program. Glob Health Action.

[14] Shanka N, Lemma S, Abyu D (2015). Recovery rate and determinants in treatment of children with severe acute malnutrition using outpatient therapeutic feeding program in Kamba District, South West Ethiopia. J Nutr Disord Ther.

[15] Badeso MH, Ferede HA, Bogale NB, Kalil FS (2019). Trends of severe acute malnutrition morbidity and mortality (2014–2017), Bale Zone, Oromia Region, Ethiopia, 2018.

[16] Abate BB, Tilahun BD, Kassie AM, Kassaw MW (2020). Treatment outcome of Severe Acute Malnutrition and associated factors among under-five children in outpatient therapeutics unit in Gubalafto Wereda, North Wollo Zone, Ethiopia, 2019. PLoS One.

[17] Desyibelew HD, Bayih MT, Baraki AG, Dadi AF (2020). The recovery rate from severe acute malnutrition among under-five years of children remains low in sub-Saharan Africa. A systematic review and meta-analysis of observational studies. PLoS One.

[18] Budul AB, Farah AM, Nour TY (2020). Treatment Outcome of Severe Acute Malnutrition Among Children (6-59 Months) in Outpatient Therapeutic Feeding Program in Gursum District, Somali Region, Ethiopia. Science.

[19] Mamo WN, Derso T, Gelaye KA, Akalu TY (2019). Time to recovery and determinants of severe acute malnutrition among 6–59 months children treated at outpatient therapeutic programme in North Gondar zone, Northwest Ethiopia: a prospective follow up study. Ital J Pediatr.

[20] Gebremedhin K, Ayele G, Boti N, Andarge E, Fikadu T (2020). Predictors of time-to-recovery from severe acute malnutrition treated in an outpatient treatment program in health posts of Arba Minch Zuria Woreda, Gamo zone, Southern Ethiopia: A retrospective cohort study. PLoS One.

[21] Association S (2018). Sphere handbook: humanitarian charter and minimum standards in humanitarian response: Practical Action.

[22] Kabthymer RH, Gizaw G, Belachew T (2020). Time to Cure and Predictors of Recovery Among Children Aged 6–59 Months with Severe Acute Malnutrition Admitted in Jimma University Medical Center, Southwest Ethiopia: A Retrospective Cohort Study. Clin Epidemiol.

[23] Massa D, Woldemichael K, Tsehayneh B, Tesfay A (2016). Treatment outcome of severe acute malnutrition and determinants of survival in Northern Ethiopia: a prospective cohort study. Int J Nutr Metabolism.

[24] Mbaya D (2015). Outcomes of severely malnourished children aged 6–59 months on outpatient management program in Kitui County Hospital.

[25] Bitew ZW, Alemu A, Worku T (2020). Treatment outcomes of severe acute malnutrition and predictors of recovery in under-five children treated within outpatient therapeutic programs in Ethiopia: a systematic review and meta-analysis. BMC Pediatr.

[26] Trehan I, Goldbach HS, LaGrone LN, Meuli GJ, Wang RJ, Maleta KM, Manary MJ (2016). Research Article (New England Journal of Medicine) Antibiotics as part of the management of severe acute malnutrition. Malawi Med J.

[27] Baraki AG, Akalu TY, Wolde HF, Takele WW, Mamo WN, Derseh B, Desyibelew HD, Dadi AF (2020). Time to recovery from severe acute malnutrition and its predictors: a multicentre retrospective follow-up study in Amhara region, north-west Ethiopia. BMJ Open.

[28] Irena AH, Mwambazi M, Mulenga V (2011). Diarrhea is a major killer of children with severe acute malnutrition admitted to inpatient set-up in Lusaka, Zambia. Nutr J.

[29] Adnyana MLD, Sidiartha IGL, Pratiwi IGAE (2020). Comorbid Diseases Is a Predictor Length of Stay in Children with Severe Acute Malnutrition. Am J Pediatr.

