# Supplementary material for: Predictors of time to recovery from uncomplicated severe acute malnutrition among 6–59 months children treated in out patient treatment in health posts of Nagele Arsi district: a retrospective cohort study
Source: BMC Pediatr. 2022 Dec 13;22:712. doi: 10.1186/s12887-022-03767-4 (PMC9746122; doi:10.1186/s12887-022-03767-4)
Supplement: Supplementary file 1 — Additional file 1. English version of the checklist used to extract information. [file 12887_2022_3767_MOESM1_ESM.docx]

**English version of the checklist used to extract information**

`Guide to data collectors: All records of children with uncomplicated severe acute malnutrition that treated on the OTP at health posts Nagele Arsi district from July 1, 2018 to June 30, 2020 are study population. Read the variables and fill the data abstraction form carefully by making appropriate circle or writing the response on the space provided accordingly in given period.

Date of admission to OTP-----------dd/----------mm/-----------------yyyy

Date of discharge from OTP---------dd/----------mm/-----------------yyyy

Name of health post------------------------- unique SAM unique NO --------------------------

| S.N | **Part I: Patient characteristics** | | | | | | | **Skip to question** |
| --- | --- | --- | --- | --- | --- | --- | --- | --- |
| 101 | Age at admission | | ____________Months | | | | |  |
| 102 | Sex | | 1. 1.Male 2. 2.Female | | | | |  |
| 103 | Distance to health post | | 1. 1. Less than 30 minutes 2. 2.More than 30 minutes 3. _____________ in minutes | | | | |  |
| 104 | Breast feeding | | 1. 1.Yes 2. 2.No | | | | |  |
| 105 | Type of admission | | 1. 1.New 2. 2.Re-admission | | | | |  |
| **Part II: Anthropometric characteristics** | | | | | | | | |
| 201 | Weight at admission | | ______________ (kg) | | | | |  |
| 202 | Target weight for non edematous | | ______________kg | | | | |  |
| 203 | Weight at discharge | | _____________kg) | | | | |  |
| 204 | MUAC at admission | | ____________ (mm) | | | | |  |
| 205 | MUAC at discharge | | ____________ (mm) | | | | |  |
| **Part III: Co-morbidities during**  **admission** | | | | | | | | |
| 301 | Admission criteria | | | | 1. 1.MUAC <11.5 2. 2.Edema grade one(+) 3. 3.Edema grade two(++) | |  | |
| 302 | Does the child have fever during admission? | | | | 1. 1.Yes 2. 2.No | |  | |
| 303 | Does the child have Malaria during admission? | | | | 1. 1.Yes 2. 2.No | |  | |
| 304 | Does the child have cough during admission? | | | | 1. 1.Yes 2. 2.No | |  | |
| 305 | Does the child have diarrhea during admission? | | | | 1. 1.Yes 2. 2.No | | IF NO 307 | |
| 306 | If yes, which type? | | | | 1. 1.Watery diarrhea 2. 2. Bloody diarrhea | |  | |
| 307 | Does the child have vomiting during admission? | | | | 1. 1.Yes 2. 2.No | |  | |
| 308 | Does the child have anemia during admission? | | | | 1. 1.Yes 2. 2.No | |  | |
| **Part IV: Routine medication** | | | | | | | | |
| 401 | | Was Vitamin A given? | | 1. 1.Yes 2. 2.No | |  | | |
| 402 | | Was Folic acid given? | | 1. 1.Yes 2. 2. No | |  | | |
| 403 | | Was de-worming given? | | 1. 1.Yes 2. 2.No 3. 3.Not applicable | |  | | |
| 404 | | Was Antibiotics given? | | 1. 1.Yes 2. 2.No 3. 3.If yes specify ----------- | |  | | |
| 405 | | Was Measles vaccine given? | | 1. 1.Yes 2. 2.No | |  | | |
| 406 | | Was Anti-malarial given? (If malaria detected) | | 1. 1.Yes 2. 2.No   3.Not applicable | |  | | |
| **Part V: Follow up** | | | | | | | | |
|  | | Weeks of follow up | | | | | | |

|  |  |  | | | | | | | | remark |
| --- | --- | --- | --- | --- | --- | --- | --- | --- | --- | --- |
| 501 | Weight |  |  |  |  |  |  |  |  |  |
| 502 | MUAC (cm) |  |  |  |  |  |  |  |  |  |
| 503 | Edema 1. ++   1. 2. + 2. 3. No |  |  |  |  |  |  |  |  |  |
| 504 | General danger sign 1. No   1. 2. Yes |  |  |  |  |  |  |  |  |  |
| 505 | Diarrhea   1. 1. No 2. 2. Yes |  |  |  |  |  |  |  |  |  |
| 506 | Vomiting   1. 1. No 2. 2. Yes |  |  |  |  |  |  |  |  |  |
| 507 | Fever 1. No 2.Yes |  |  |  |  |  |  |  |  |  |
| 508 | Cough 1.No   1. 2.Yes |  |  |  |  |  |  |  |  |  |
| 509 | Anemia(Palmar pallor)   1. 1. No   2.Yes |  |  |  |  |  |  |  |  |  |
| 510 | Skin infection   1. 1. No 2. 2. Yes |  |  |  |  |  |  |  |  | If no skip to 512 |
|  | 1. (+,++) |  |  |  |  |  |  |  |  |  |
| 511 | Appetite test during follow up,   1. 1. Fail 2. Pass |  |  |  |  |  |  |  |  |  |
| 601 | Out come | | | | 1. 1.Cured 2.Defaulter 3.Transferred 2. 4 Non respondent 5. Death 3. 6. Unknown | | | | |  |
| 602 | How many days did the patient stayed in the program? | | | | ------------ days | | | | |  |

**Thank you**
